# Early Intervention Referral Rates of Infants With Neonatal Opioid Withdrawal Syndrome Are Not Significantly Affected by Race, Payor, Maternal Medication for Opioid Use Disorder, or NICU Hospitalization

**DOI:** 10.7759/cureus.83559

**Published:** 2025-05-06

**Authors:** Weijen Chang, Laura Lee, Samantha Roberge, Madeline Chang, Elizabeth Peacock-Chambers

**Affiliations:** 1 Pediatrics, Baystate Medical Center, Springfield, USA; 2 Pediatrics, MaineGeneral Health, Augusta, USA; 3 Pediatric Endocrinology, Cincinnati Children's Hospital Medical Center, Cincinnati, USA; 4 Brooks School of Public Policy, Cornell University, Ithaca, USA

**Keywords:** early intervention, neonatal abstinence syndrome, neonatal intensive care unit, neonatal opioid withdrawal syndrome, pediatric hospital medicine

## Abstract

In this study, we examined the factors influencing enrollment in early intervention (EI) programs for neonates diagnosed with nonopioid-associated neonatal abstinence syndrome (NAS) and neonatal opioid withdrawal syndrome (NOWS). The primary goal was to examine characteristics linked to completing an individualized family service plan (IFSP), marking EI enrollment. Patient records were reviewed using data from neonates discharged with NOWS/NAS at a Massachusetts hospital in 2017 to identify demographic and medical factors related to NOWS/NAS cases. Out of 125 cases, 111 were analyzed, with patient outcomes analyzed in subgroups of those with NOWS and non-opioid-associated NAS. Findings indicated that neonates with NICU hospitalization, race, payor, and maternal medication for opioid use disorder (MOUD) did not have significantly different rates of IFSP completion. Additionally, no significant difference was observed in IFSP completion rates between neonates with NAS or NOWS. This study reports baseline data from a quality improvement initiative not designed to detect statistically significant differences. However, it highlights potential areas for further investigation to improve EI access for NOWS- and NAS-affected neonates, although more extensive studies are needed to further elucidate these differences.

## Introduction

Neonatal abstinence syndrome (NAS) was the original term given to a syndrome observed when an infant was exposed to licit or illicit chemical substances in utero and manifested symptoms of withdrawal from the substance(s) after birth [1]. While NAS is a general term for symptoms seen in the newborn period associated with in utero exposure to various substances, neonatal opioid withdrawal syndrome (NOWS) is a subset of NAS and refers specifically to withdrawal from maternal opioid exposures [2]. A prior 2019 study showed that fewer than half of eligible infants with NAS ultimately enrolled in EI services, highlighting a gap in processes leading to early intervention (EI) enrollment [3]. This is despite the fact that NAS and NOWS are automatically qualifying diagnoses for up to one year of EI services. Appropriate identification, documentation, and communication of NOWS and NAS diagnoses are needed to qualify for federally funded therapies to support development through EI programs [4]. Barriers to EI referral and enrollment have been identified, including inadequate inpatient documentation, parental health beliefs, and psychosocial stressors [5,6]. Other characteristics associated with lower rates of EI enrollment in a prior study included Black race and Hispanic ethnicity [7]. Our objective is to assess whether we could identify characteristics associated with successful EI referral and enrollment in infants discharged with NOWS and whether EI enrollment rates differ between NOWS and non-opioid-associated NAS. Successful EI referral and enrollment were indicated by a record of completing an individualized family service plan (IFSP) in data provided by the Massachusetts Department of Public Health.

The content of this article was previously presented in meeting abstracts at the 2020 Society of Hospital Medicine Converge meeting (April 9, 2020) and the Hospital Medicine 2019 Conference (March 26, 2019).

## Materials and methods

We started with an initial analysis of whether the International Classification of Diseases 10th Revision (ICD-10) codes associated with birth hospitalizations could be used to identify cases of NOWS and nonopioid-associated NAS. From January to March 2017, all discharges of neonates born at our 107-bed children’s hospital were identified utilizing a billing database. A total of 922 discharges were identified, 139 from the neonatal intensive care unit (NICU) and 783 from the newborn nursery (NBN). Hospital documentation of all discharged neonates was reviewed manually to identify babies who were exposed to substances associated with NAS/NOWS and subsequently developed withdrawal symptoms, as outlined in the 2012 American Academy of Pediatrics Clinical Report [1].

A financial analyst's review of billing databases identified neonates with ICD-10 codes commonly utilized to designate NOWS and NAS (Table 1).

**Table 1:** ICD-10 codes and their corresponding diagnoses related to neonatal conditions caused by maternal substance use International Classification of Diseases 10th Revision (ICD-10) code: Standardized medical coding system for diagnoses. Diagnosis associated with the ICD-10 codes: Describes neonatal conditions influenced by maternal exposure to various substances during pregnancy, labor, or delivery. Codes P04.0–P04.9 refer to newborns affected by maternal exposure to anesthesia, medications, tobacco, or addictive substances. P96.1 specifies neonatal withdrawal symptoms due to maternal drug use.

ICD-10 code	Diagnosis associated with ICD-10 code
P04.0	NB aff by matern anesth and analgesia in preg, labor and del
P04.1	Newborn affected by other maternal medication
P04.2	Newborn affected by maternal use of tobacco
P04.41	Newborn affected by maternal use of cocaine
P04.49	Newborn affected by maternal use of other drugs of addiction
P04.8	Newborn affected by other maternal noxious substances
P04.9	Newborn affected by maternal noxious substance, unspecified
P96.1	Neonatal w/drawal symp from matern use of drugs of addiction

The initial analysis of all birth hospitalizations from January to March showed that of 22 babies confirmed as having NOWS through manual chart review, five of seven discharged from the NBN and 15 of 15 discharged from the NICU were assigned a billing code associated with in utero opioid exposure, resulting in sensitivities of 71.4% and 100%, respectively. Of 63 babies confirmed as exposed to NAS due to non-opioid substances (including tobacco, alcohol, cannabinoids, antidepressants, cocaine), through chart review, 12 of 54 discharged from NBN, and nine of nine discharged from NICU were assigned a billing code associated with in utero drug exposure, resulting in sensitivities of 22.2% and 100%, respectively. Two babies with no identified NAS-associated substance exposure (one from NICU and one NBN each) were assigned billing codes associated with in-utero drug exposure, resulting in specificities of 99.8% and 99.1%, respectively [8].

**Figure 1:**
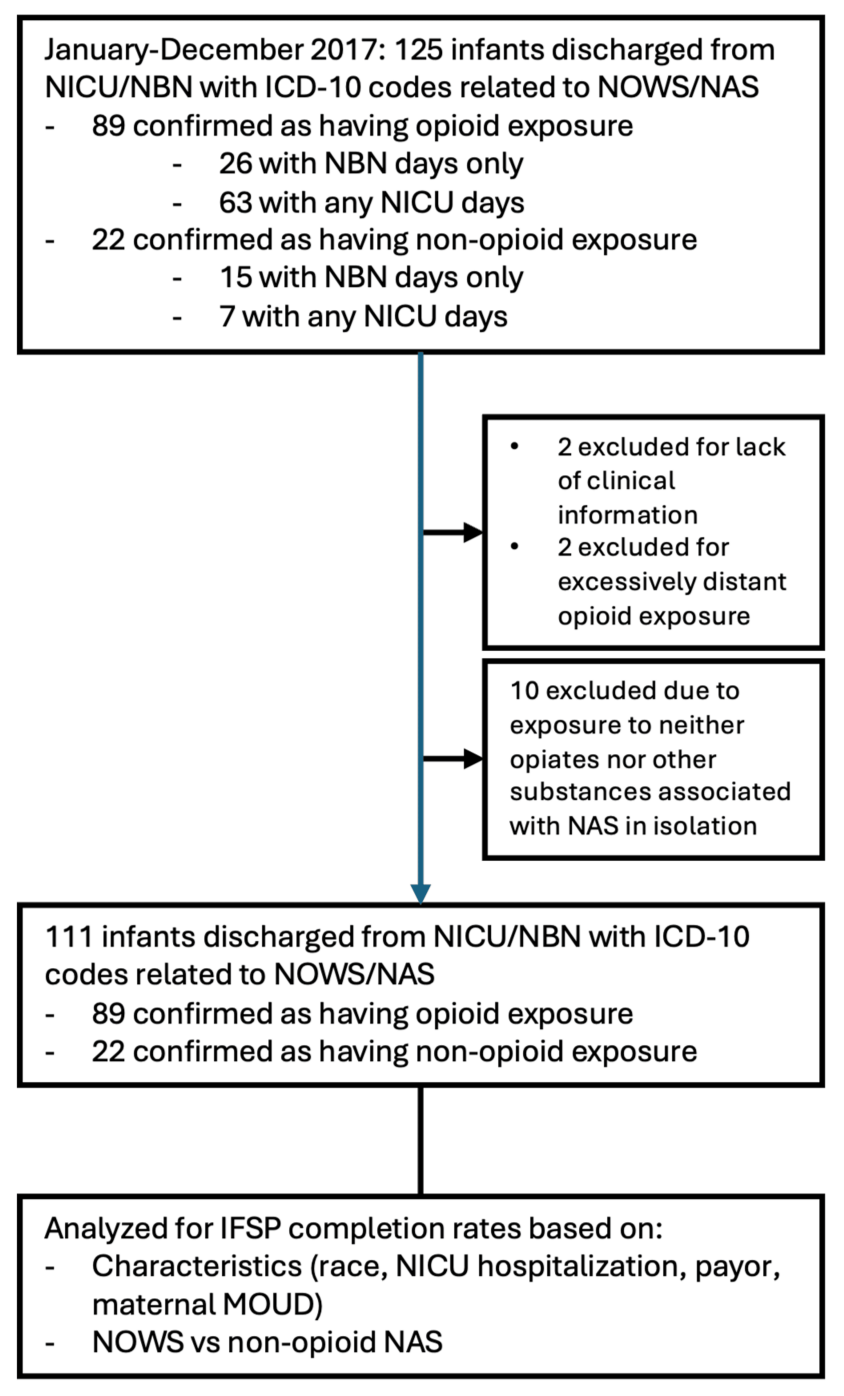
Cohort selection for the analysis of early intervention completion among infants with NAS/NOWS This flow diagram depicts the selection and refinement of a study cohort consisting of infants discharged from the Neonatal Intensive Care Unit (NICU) or Newborn Nursery (NBN) between January and December 2017 with International Classification of Diseases, 10th Revision (ICD-10) codes related to neonatal abstinence syndrome (NAS) or neonatal opioid withdrawal syndrome (NOWS). Among the 125 infants initially identified, 89 were confirmed to have opioid exposure, and 22 had exposure to non-opioid substances associated with NAS. Infants were further categorized based on whether they received care exclusively in the NBN or had any NICU days. A total of 14 infants were excluded due to insufficient clinical information (n = 2), excessively distant timing of opioid exposure (n = 2), or no documented exposure to opioids or other substances known to cause NAS in isolation (n = 10). This resulted in a final cohort of 111 infants—89 with opioid exposure and 22 with non-opioid exposure. These infants were subsequently analyzed for completion of an individualized family service plan (IFSP), based on clinical and demographic characteristics, including race, NICU hospitalization, insurance payor type, and maternal receipt of medication for opioid use disorder (MOUD). IFSP completion rates were also analyzed based on type of withdrawal syndrome (NOWS vs. non-opioid NAS).

Confirmation of high levels of specificity of NOWS- and NAS-associated billing codes overall and high sensitivity in the NICU setting allowed identification of potential cases based on ICD-10 analysis to proceed for January-December 2017 (Figure 1). Low sensitivities of NOWS- and NAS-associated billing codes in the NBN setting likely led to missed cases in the analysis of NBN data. Among the 125 infants initially identified, 89 were confirmed to have opioid exposure, and 22 had exposure to non-opioid substances associated with NAS. Infants were further categorized based on whether they received care exclusively in the NBN or had any NICU days. A total of 14 infants were excluded due to insufficient clinical information (n = 2), excessively distant timing of opioid exposure (n = 2), or no documented exposure to opioids or other substances known to cause NAS in isolation (n = 10). This resulted in a final cohort of 111 infants, 89 with opioid exposure and 22 with non-opioid exposure.

Data from the neonatal discharges described above were then correlated to data supplied by the Massachusetts Department of Public Health for rates of completion of an IFSP. Completing an IFSP was considered a proxy for enrollment in EI, as it is a foundational document in EI enrollment. The IFSP is designed to support infants at risk for developmental delays and is developed through a collaborative process between the family and a multidisciplinary team [4]. A single reviewer confirmed the clinical diagnosis and assessed each patient for characteristics documented in the electronic medical record (EMR), including stated race (White, Black, Asian), Hispanic or non-Hispanic, hospitalization in the NICU at any time during the birth hospitalization, maternal treatment with medication for opioid use disorder (MOUD) with maintenance opioid (methadone, buprenorphine, or buprenorphine-naloxone), and payor (commercial or government). IFSP completion rates were also analyzed based on type of withdrawal syndrome (NOWS vs. non-opioid NAS).

The Institutional Review Board determined this work to be non-human subjects research based on its use as baseline data collection for an ongoing quality improvement effort.

This study was part of a quality improvement (QI) initiative, and as such, a formal power calculation was not performed prior to data collection. The primary aim was to identify patterns and potential associations that could inform future interventions and systems-level changes. Chi-square tests of independence were used to explore associations between categorical variables and completion of an IFSP, a proxy for early intervention (EI) enrollment. This statistical approach is appropriate in the QI context, as it allows for exploratory assessment of trends within existing data, even when not powered for hypothesis testing. Results are reported with corresponding chi-square values and p-values for transparency.

## Results

Among neonates with opiate-associated NAS (Table 2), chi-square tests of independence were performed to examine the association between IFSP completion and four patient or maternal characteristics: NICU hospitalization during the neonatal course, stated race, presence of a commercial payor, and maternal treatment with MOUD. While higher rates of IFSP completion were observed among those with NICU hospitalization (19 vs. 32), Black race (4 vs. 6), commercial payor (6 vs. 5), and maternal MOUD (16 vs. 20), none of these differences reached statistical significance (NICU hospitalization: χ²(1, n = 81) = 0.47, p = 0.49; Black race: χ²(1, n = 81) = 0.00, p = 1.00; commercial payor: χ²(1, n = 81) = 0.35, p = 0.55; maternal MOUD: χ²(1, n = 81) = 0.04, p = 0.84).

**Table 2:** Characteristics of infants with neonatal opioid withdrawal syndrome (NOWS), with and without IFSP completion This table compares infant characteristics based on the completion of an individualized family service plan (IFSP), with data presented for those who completed (n = 24) and did not complete (n = 65) the IFSP. It includes the percentage of infants who experienced neonatal intensive care unit (NICU) hospitalization, were identified as Black race, had commercial insurance, and whose mothers received medication for opioid use disorder (MOUD) during pregnancy.

Characteristics	IFSP completed (%), n=24	IFSP not completed (%), n=65
NICU hospitalization	19 (79.2)	43 (66.2)
Black race	3 (12.5)	4 (6.2)
Commercial payor	6 (25.0)	9 (13.8)
Maternal MOUD	21 (87.5)	55 (84.6)

To evaluate whether the type of substance exposure (opiate-associated vs. non-opiate-associated NAS) was associated with IFSP completion (Table 3), a separate chi-square test was conducted. Among neonates with opiate-associated NAS, 35 (39%) completed an IFSP, compared to six (27%) among those with non-opiate-associated NAS. This difference was not statistically significant (χ²(1, n = 111) = 0.64, p = 0.42).

**Table 3:** Rates of individualized family service plan (IFSP) completion in neonatal opioid withdrawal syndrome (NOWS) vs. non-opioid neonatal abstinence syndrome (NAS) This table compares the completion status of an individualized family service plan (IFSP) between infants diagnosed with non-opioid neonatal abstinence syndrome (NAS) (n = 22) and those with neonatal opioid withdrawal syndrome (NOWS) (n = 89). The data are presented as the number and percentage of infants who completed or did not complete an IFSP within each diagnostic group.

Characteristics	Non-opioid NAS (%), n = 22	NOWS (%), n = 89
IFSP completed	7 (31.8)	25 (28.1)
IFSP not completed	15 (68.2)	64 (71.9)

These findings suggest that, although certain characteristics were observed to have higher rates of EI referral and enrollment among infants with NAS, the observed differences were not statistically significant in this sample. The analysis was likely underpowered to detect modest differences due to the small sample size. This analysis should be considered an exploratory evaluation, rather than a confirmatory hypothesis test.

## Discussion

In our initial methodologic analysis of ICD-10 code accuracy in NAS/NOWS, we found that, while analysis of administrative billing data is not sensitive in the newborn nursery setting, it proved to be specific enough to be relied upon for NAS and NOWS identification. We conjectured that the lower sensitivity of ICD-10 codes associated with NAS and NOWS in the newborn nursery may be due to the higher turnover and more templated documentation in this setting compared to the NICU. Other studies looking at the accuracy of administrative billing codes in NAS and NOWS include a study by Maalouf et al., who found a positive predictive value (PPV) of administrative data for NAS using Tennessee Medicaid claims to be 91% for ICD-9 codes and 98.2% for ICD-10 codes [9]. In Massachusetts, Goyal et al. found the sensitivity for NAS using ICD-10 codes to be >79% and the PPV for the P96.1 codes to be >92% [10].

While the results of the primary objective of this study are not statistically significant and cannot be correlated with existing literature regarding EI referrals, considering other literature on this topic is instructive. A study of women with OUD in Massachusetts published in 2020 by Peeler et al. showed that non-Hispanic Black and Hispanic women were less likely to receive medication-assisted treatment during pregnancy, contributing to lower EI referral rates [11]. Dookeran et al. found in a 2023 study of the HCUP-KID national all-payer pediatric inpatient-care database that non-Hispanic White neonates have a higher prevalence of NAS, typically increasing their likelihood of referral [12]. A 2020 study by Umer et al. of newborns in West Virginia found that Medicaid covered 86% of all infants diagnosed with NAS cases [13].

More consistent with our data, NICU hospitalization is associated with high levels of EI referrals [14,15], suggesting that NICU providers and staff are experienced in documenting the need for and enabling successful referrals for EI. In addition, Barfield et al. found in a 2008 study of EI referrals in Massachusetts that infants with private insurance were more likely to be referred to EI services than those without private insurance [16]. McManus et al. also found that children with higher family income, which often correlates with having commercial insurance, were more likely to receive EI services [17]. Mothers treated for opioid use disorder with MOUD were found by Peeler et al. to have their children referred for EI [11]. Further study of regional characteristics that place newborns with NOWS and NAS at higher risk for non-completion of EI referrals is critical to appropriate target resources, as factors that would promote or hinder successful EI referrals vary regionally. A 2021 study by Young et al. showed significant site-to-site variation in prenatal care, rates of maternal MOUD, prenatal counseling, and infants receiving pharmacological therapy and secondary medications [18].

Limitations of this study include the low sensitivity of ICD-10 codes for identifying NOWS and NAS. This may have resulted in selection bias, potentially underestimating the true number of affected infants and skewing the sample toward those with more severe presentations or better-documented cases. This limitation could influence the generalizability of findings and obscure associations with early intervention outcomes. In addition, this study's limitations include the single-center, retrospective design, small sample size, and reliance on ICD-10 coding. In particular, the small sample size limits the statistical power of the analysis, increasing the risk of type II error and potentially obscuring meaningful associations between patient characteristics and early intervention outcomes. We also examined commercial versus government insurance but did not assess other key social determinants of health, such as parental education, housing stability, access to healthcare resources, or non-English language preference. In regard to IFSP completion, we did not ascertain the reasons for EI referral, which may not have been due primarily to substance exposure. These potential confounding factors should be considered in interpreting the lack of significant difference between IFSP completion in opioid and non-opioid exposed neonates.

## Conclusions

While we observed differences in successful EI enrollment, based on IFSP completion rates, associated with specific demographic and clinical characteristics, they were not statistically significant. There were no significant differences in IFSP completion between neonates with NOWS and those with non-opioid-associated NAS. These findings show the need for larger studies to fully assess the associations of the studied characteristics with successful EI enrollment. In addition, extracting and analyzing data of mother-infant dyads from the electronic medical record is now available and should improve the accuracy of data analysis.
